# Acute Pancreatitis—II

**Published:** 1912-07-27

**Authors:** 


### ACUTE PANCREATITIS—II.

### Its Diagnosis and Surgical Treatment.

In pancreatitis the development of epigastric pain occurs frequently and often early, while in obstruction there is practically never a localisation of these signs to the epigastrium. Slight jaundice often develops in pancreatitis; in obstruction it is absent. The rapid development of free fluid in the abdominal cavity would point to pancreatitis. If the case should progress for some days there is in pancreatitis the gradual appearance of signs of omental bursitis, an upper abdominal lesion, associated with a severe toxæmia, but with subsidence of the signs of ileus, vomiting, etc. In obstruction there is also a toxæmia from the absorption of stagnated bowel contents, and the signs of intestinal stasis plainly manifest themselves if continued; finally, there are fæcal vomiting, increasing signs of peritonism, marked distension, and tympany.

Acute gastro-duodenitis may possibly be confused with pancreatitis. It must, indeed, be a severe type of this illness which would simulate the graver lesion. Indeed, it seems hardly more than necessary to consider the symptoms of each to arrive at a diagnosis, should this be the differential question. Biliary colic, with or without slight jaundice, would give many points of similarity to the initial pain of pancreatitis. At the time of the onset of the pain a distinction might be possible. Gall-stone colic is far more common than acute pancreatic lesions are, and they may be associated so that the physician would think of that condition first. As a rule the location of the pain to the right of the median line, its radiation, and the failure of other pancreatic symptoms to supervene should guide one correctly. Yet it is to be remembered that in pancreatitis the location of the pain is variable, and radiation to the back and shoulders has been mentioned as at times occurring. The diagnosis would be more difficult when there is a perforative peritonitis as the result of some lesion in the biliary passages. This condition would more closely resemble other varieties of perforative peritonitis of the upper abdomen.

Other conditions possibly simulating acute pancreatitis have been poisoning, impacted ureteral calculus, etc. In these the course of events, the other and distinctive signs, and in the former the history, should soon serve to put one on the right track. Thrombosis of the mesenteric vessels, practically never diagnosed except at operation, may give rise to symptoms of ileus and peritonism, very likely to simulate acute pancreatitis. Acute appendicitis has been the diagnosis of several cases of pancreatitis. Appendicitis is, however, distinctly an inflammatory condition of the lower abdomen; its symptomatology is well marked and typical in most cases, and its signs, with few exceptions, are not those of an upper abdominal lesion.

Next to a diagnosis of acute obstruction in those cases of pancreatitis not recognised, there is that of an acute perforative peritonitis of the upper abdomen, be it of stomach, duodenum, or bile ducts. In the first two conditions the history, if obtainable, would be something of a guide, but when the biliary system is the seat of perforation the history may be misleading rather than a help. In all these conditions the sudden agonising pain in the epigastrium would at once point to a serious lesion of the upper abdomen; indeed, it more often happens that the closest approach to the diagnosis that one can arrive at is that of an acute abdominal lesion requiring laparotomy, and the diagnosis is at once obvious when the typical islands of fat necrosis are found in the omental fat. Dr. Deaver, Surgeon-in-Chief to the German Hospital, Philadelphia, has been fortunate enough to meet with a good many cases of the disease which he has recorded, including three in which recovery occurred after operation. His views as to the diagnosis of the condition are given above, and his opinions as to the treatment of acute pancreatitis are as follows:—

But a few years ago pancreatitis, except when progressing to suppuration, or the formation of a palpable mass, was considered to belong to the field of medical treatment alone. Surgical intervention was considered hazardous and almost useless. Several cases have been reported as recovering without operation, in which there were apparently the symptoms of an acute pancreatic lesion. Needless to say, recovery under these circumstances is rare; indeed, it may truthfully be stated that the chance of such a non-operative recovery is comparatively slight.

The medical treatment of acute pancreatitis has been aptly compared to a similar procedure in acute appendicitis. The latter affection was beyond the range of surgery many years after its pathology was well known. It may be admitted then that the treatment of an acute pancreatitis is by surgery. This being so, there are three important features to be considered as regards operation: (1) At what stage of the illness should the patient be operated upon? (2) What method of approach to the seat of disease shall be adopted? (3) What should be the technique after reaching the pancreas or the pancreatic abscess?

1. *The stage at which operation should be undertaken* in acute pancreatitis varies with the variety of the disease. In a fulminating ultra-acute case, the rapid progress of the patient from bad to worse may make early operation necessary. Operation should not be undertaken in the state of primary shock, following the onset of the distinctive symptoms of pancreatitis. Cases in which this has been done have almost invariably come to a fatal termination. The tendency among surgeons who have investigated the question, and have had the opportunity of seeing a number of cases, seems to be to operate comparatively early. Nevertheless, Dr. Deaver is convinced that this rule does not always hold good. Thus, in one of his cases which terminated as a gangrenous pancreatitis, with sloughing of nearly the whole organ, it is almost certain that surgical intervention in the first few days would have ended in the patient's death. Delay with stimulation allowed him to operate upon a patient in somewhat improved condition, and to make use of localising signs to approach the pancreas by a much more favourable route than it would have been possible to adopt had immediate operation been undertaken. It must be understood, then, that while in cases becoming rapidly and progressively worse, operation may be imperative, it is generally of advantage to give the organism an opportunity to prepare itself, so to speak, for a still greater tax upon it. Moreover, when there is delay until an abscess extending into the loin has been formed, one is able to approach it through the loin.

2. *The Method of Approach to the Seat of Disease.*—The routes to the pancreas may be grouped under two main heads: (1) those which involve the traversing of the general peritoneal cavity, and (2) the extraperitoneal route by the loin incision.

(1) When there is a beginning acute pancreatitis, when the localising symptoms are all epigastric, when there is a tumour palpable anteriorly, giving a tympanitic note on percussion and a sense of resiliency on palpation, and when there is doubt concerning the diagnosis, one is compelled to make the incision upon the anterior aspect of the abdomen, and to traverse the general peritoneal cavity in order to reach the pancreas. Once in the general peritoneal cavity the pancreas itself can be approached either (*a*) through the lesser omentum, (*b*) through the gastro-colic omentum, or (*c*) through the transverse mesocolon. As a general rule the anterior or intraperitoneal incision has been adopted by almost all operators. It has one serious disadvantage—that is, the risk of infecting the general peritoneal cavity. Its advantages are the free exposure of the operative field, opportunity for radical surgery, and the opportunities which it offers for the establishment of adequate drainage. The choice of a path above or below the stomach must depend upon circumstances. When it is necessary to secure an extra path for drainage, tampons and drains must be carried both above and below the stomach.

(2) The extraperitoneal route, practically as for exposure of the kidney, allows approach to the pancreas, and especially the tail thereof, without entering the general peritoneal cavity. The chief advantages of the extraperitoneal route when feasible cannot be questioned, except possibly by a few enthusiasts who, by preference, open appendical abscesses and remove ureteral stones through the peritoneal cavity. Naturally it is only applicable in those instances in which there is a distinct indication for it; that is, the appearance of symptoms pointing to the localisation of inflammatory exudate or pus in the loin. By it good drainage is obtainable for the pancreas and the omental bursa. Its disadvantages are, however, many. There is no free exposure of the parts, and radical surgery is impossible; drainage is possible, and that is all. Gall-stones or concomitant gastric lesions, etc., are entirely inaccessible by this method. Two cases of Dr. Deaver's series were operated upon by the anterior incision, and correctly so, as in each case the localising symptoms demanded the opening of the greater peritoneal cavity. In another case, after an operation for gall-stones had been carried out, the post-pancreatic collection was evacuated by the loin. Thus the danger of peritonitis was avoided, and at the same time the underlying biliary condition was adequately dealt with.

In this connection he mentions the fact that in some of the reported instances of acute gangrenous or suppurative pancreatitis drainage has been instituted by both the anterior and posterior routes. In one of his cases there was an omental bursitis with distinct localising signs in the left loin. Here it was deemed better to undertake the unmistakably less serious extraperitoneal operation, rather than to ignore the symptom which clearly indicated the correct avenue of approach to the seat of the lesion. He believes that he would not have achieved an equally favourable outcome by any other operative procedure. The wound drained freely, the pancreas as it sloughed was discharged without difficulty, and the only unsatisfactory feature was the impossibility of obtaining a fully exposed wound when dressing it.

3. *The Technique after Reaching the Pancreas.*—Whether one has to deal with a pancreatitis still in the presuppurative or hæmorrhagic stage, or whether pus has already formed, there is but one cardinal indication for treatment—the establishment of adequate drainage.

Should it be found that there is an acute hæmorrhagic pancreatitis, or an acute gangrenous pancreatitis, having approached the organ by the intraperitoneal route, the only possible procedure locally is to apply tampons and drains freely to the organ itself. Dr. Deaver is of opinion that tube drainage is always essential. Gauze alone will do more to prevent than to establish drainage.

He holds that when one finds an engorged and inflamed pancreas it is expedient to incise the capsule and apply drains to this exposed surface. Certain recent investigators, however, have come to the conclusion that this incision into the organ itself is unnecessary. Noetzel, especially, who has written the most recent résumé on the subject takes this view. He brings strong support for his argument from his own experience and that of others. But the question is still open.

It is practically never possible to resect the pancreas satisfactorily for inflammatory lesions, and this is, indeed, unnecessary. Drainage gives free exit to the debris.

Should there be any free fluid in the peritoneal cavity it will be necessary to see to its removal. It should not be forgotten that in this pancreatic exudate toxic material is present often in a sufficient quantity to produce death. In this connection, it is interesting to note that in one of the first cases of this kind reported, that of Halsted, the evacuation of the free fluid in the peritoneal cavity was the only thing attempted. The patient recovered, and while it may be true, as Korte has remarked concerning this case, that the patient recovered in spite of and not because of the operation, yet it is doubtful if recovery would have taken place with any added toxins for him to overcome.

(*To be continued.*)

